# The Effect of Neurotoxin MPTP and Neuroprotector Isatin on the Profile of Ubiquitinated Brain Mitochondrial Proteins

**DOI:** 10.3390/cells7080091

**Published:** 2018-07-31

**Authors:** Olga Buneeva, Arthur Kopylov, Inga Kapitsa, Elena Ivanova, Victor Zgoda, Alexei Medvedev

**Affiliations:** 1 Department of Proteomic Research and Mass Spectrometry, Institute of Biomedical Chemistry, 10 Pogodinskaya Street, Moscow 119121, Russia; olbuneeva@gmail.com (O.B.); a.t.kopylov@gmail.com (A.K.); victor.zgoda@gmail.com (V.Z.); 2 Zakusov Institute of Pharmacology, 8 Baltiskaya Street, Moscow 124315, Russia; ingakap73@main.ru (I.K.); iwanowaea@yandex.ru (E.I.)

**Keywords:** ubiquitin, proteasome, mouse brain mitochondrial proteins, proteomic profiling of ubiquitinated proteins, MPTP-induced Parkinsonism, neuroprotector isatin

## Abstract

Mitochondria are a crucial target for the actions of neurotoxins, causing symptoms of Parkinson’s disease in various experimental animal models, and also neuroprotectors. There is evidence that mitochondrial dysfunction induced by the neurotoxin 1-methyl-4-phenyl-1,2,3,6-tetrahydropyridine (MPTP) influences functioning of the ubiquitin-proteasomal system (UPS) responsible for selective proteolytic degradation of proteins from various intracellular compartments (including mitochondria) and neuroprotective effects of certain anti-Parkisonian agents (monoamine oxidase inhibitors) may be associated with their effects on the UPS. In this study, we have investigated the effect of the neurotoxin MPTP and neuroprotector isatin, and their combination on the profile of ubiquitinated brain mitochondrial proteins. The development of movement disorders induced by MPTP administration caused dramatic changes in the profile of ubiquitinated proteins associated with mitochondria. Pretreatment with the neuroprotector isatin decreased manifestations of MPTP-induced Parkinsonism, and had a significant impact on the profile of ubiquitinated mitochondrial proteins (including oxidative modified proteins). Administration of isatin alone to intact mice also influenced the profile of ubiquitinated mitochondrial proteins, and increased the proportion of oxidized proteins carrying the ubiquitination signature. These alterations in the ubiquitination of mitochondrial proteins observed within 2 h after administration of MPTP and isatin obviously reflect immediate short-term biological responses to these treatments.

## 1. Introduction

Mitochondria play an important role in molecular mechanisms of adaptive changes occurring in cells of various brain structures in response to altered physiological conditions or development of pathologies [1]. These intracellular organelles are a crucial target for various neurotoxins, causing symptoms of Parkinson’s disease [1,2,3]. In the context of experimental Parkinsonism induced by administration of 1-methyl-4-phenyl-1,2,3,6-tetrahydropyridine (MPTP) mitochondria are the principle organelles, where the key events in the development of this neurodegenerative disorder occur. Being a protoxin, MPTP undergoes bioactivation by monoamine oxidase B (MAO B), the enzyme of the outer mitochondrial membrane; the resultant neurotoxin MPP^+^ (1-methyl-4-phenylpyridinium) inhibits complex I of the mitochondrial respiratory chain, thus promoting the development of mitochondrial dysfunction and movement disorders typical of Parkinson’s disease [3,4,5]. Administration of MAO B inhibitors (e.g., deprenyl or isatin [6,7]) or substrates competing for the active site of this enzyme (e.g., phenylethylamine) [8] prevented not only metabolic activation of MPTP, but also deficiency of the neurotransmitter dopamine, and locomotor impairments typical of this disease.

Good evidence exists in the literature that neuroprotector mechanisms of certain anti-Parkisonian agents, inhibitors of monoamine oxidase (MAO), may be associated with their effects on the ubiquitin-proteasomal system (UPS) [9] responsible for selective proteolytic degradation of proteins from various intracellular compartments including mitochondria. Mitochondrial dysfunction induced by MPTP has a significant impact on the functioning of the ubiquitin-proteasome system (UPS) [10,11].

Ubiquitin, a 76-residue protein, is widely distributed in all eukaryotic cells, and targets proteins for subsequent degradation [12,13,14]. The ubiquitination process includes several sequential stages, which involve ubiquitin-activating enzyme (E1), ubiquitin-conjugating enzyme (E2), and ubiquitin ligase (E3) [12,13,14]. The major function of ubiquitin consists of (poly)ubiquitination of proteins for their subsequent proteasomal degradation. In this context, the 19S proteasome subunits play a role in the ubiquitin receptor [15], which is responsible for the delivery of client proteins to the 20S proteasome, where subsequent proteolytic degradation occurs [16].

Recently, we have demonstrated that one of the 19S proteasome subunits, Rpn10, binds a wide range of mouse proteins associated with brain mitochondria [17]; and it demonstrated high affinity to both ubiquitinated and to non-ubiquitinated proteins [18]. The development of MPTP-induced movement impairments typical of experimental Parkinsonism in mice were accompanied by changes in the repertoire of Rpn10-binding proteins [17]. Pretreatment of mice with isatin significantly attenuated the manifestation of movement impairments, and decreased the repertoire of mitochondrial Rpn10-binding proteins [17]. However, the ubiquitination status of these proteins was not evaluated.

In this work, we have studied the profile of the mouse brain mitochondrial proteins carrying the ubiquitin signature and the possible contribution of cytosolic and mitochondrial ubiquitin conjugation machineries to the ubiquitination of mitochondrial proteins. We have also investigated the effect of the neurotoxin MPTP and the neuroprotector isatin on the profile of ubiquitinated proteins of the mouse brain mitochondrial fraction. The results of this study indicate that the development of MPTP-induced Parkinsonism had a significant impact on the profile of ubiquitinated brain mitochondrial proteins. Administration of the neuroprotector isatin either alone or before MPTP significantly altered the profile of ubiquitinated brain mitochondrial proteins.

## 2. Materials and Methods

### 2.1. Reagents

Sucrose, Triton X-100, triethylammonium bicarbonate, potassium phosphates, deoxycholic acid sodium salt, urea, 2-iodoacetoamide, MPTP, and isatin were purchased from Sigma (St. Louis, MO, USA); formic acid was from Merck (Darmstadt, Germany); acetonitrile was from Fisher Chemical (Leicestershire, UK); tris-(2-carboxyethyl) phosphine was obtained from Pierce-Thermo Scientific (Rockford, IL, USA). Trypsin (modified sequencing grade) was obtained from Promega (Madison, WI, USA). Deubiquitinase inhibitor degrasyn (WP-1130) was purchased from Selleck Chemicals (Houston, TX, USA).

### 2.2. Animals, MPTP, and Isatin Administration, and Behavioral Tests

Thirty two male C57BL/6 mice (20–25 g; *n* = 8 in each group), obtained from the Stolbovaya nursery (Moscow region), were used in this study. Experiments were performed one week after their arrival from the nursery. Animals were maintained at natural illumination and received a standard laboratory chow and water ad libitum. MPTP was injected intraperitoneally (i.p., 30 mg/kg). Isatin (100 mg/kg, i.p.) was injected 30 min before MPTP. Control mice were treated with intraperitoneal injection of saline (0.1 mL/kg). Behavioral changes induced by MPTP or isatin were analyzed 90 min after the last administration by means of the open field test [19]. The exploratory reaction of mice in the open field test was defined as a sum of horizontal activity (units) and vertical activity (units). All procedures were approved by local authorities for animal research.

### 2.3. Isolation of Mitochondrial Fraction and Sample Preparation for Mass Spectrometry

The animals were decapitated within 30 min after behavioral testing. All subsequent procedures were carried out at 4 °C. The brains, washed in ice-cold saline, were immediately dissected and homogenized in the isolation medium containing 0.32 M sucrose, 1 mM EDTA, 10 mM Tris-HCl buffer, pH 7.5, using an Ultra-Turrax T 10 homogenizer (IKA-Werke, Staufen Germany) at a low speed, to obtain 10% homogenate (*w*/*v*).

Isolation of the brain mitochondrial fraction was carried out as described previously [20]. Briefly, 10% homogenate was initially centrifuged at 1000× *g* for 10 min to remove sediment cell debris and nuclei. The resultant supernatant was further centrifuged at 10,000× *g* for 20 min to isolate the crude mitochondrial fraction, which was not subjected to additional purification procedures.

The mitochondrial pellets obtained at the previous stage were resuspended in 200 µL in the lyzing solution, containing 0.05 M potassium-phosphate buffer, pH 7.4, and 3% Triton X-100. After incubation for 60 min at 4 °C, the mitochondrial preparations were diluted three times with the same buffer but without Triton X-100 to obtain the final concentration of the detergent of 1%. Samples were centrifuged at 19,500× *g* for 20 min, and supernatants were used for subsequent proteomic analysis [21].

In a pilot experiment, the lysates were incubated with the deubiquitinating enzyme inhibitor degrasyn (WP-1130) for the evaluation of a possible effect of endogenous deubiquitinases on ubiquitinated proteins.

### 2.4. Mass Spectrometry and Liquid Chromatography

High resolution mass spectrometry analysis was performed using an Orbitrap Fusion (Thermo Scientific, Rockford, IL, USA) with the installed ESI-NSI ion source. The instrument was operated in positive ionization mode with emitter voltage adjusted to 2.2 kV and drying gas temperature at 280 °C. Surveyed in a range of 400 *m/z*–1200 *m/z* precursor ions (maximum integration time was 80 ms) with charge states from z = 2+ to z = 6+ were isolated in the quadrupole mass analyzer within ±1.5 *m*/*z*, and triggered to fragmentation in the *m/z* range with a fixed lower mass (110 *m/z*) and a dynamic upper mass (depending on the charge state of the fragmented precursor) limited to 2100 *m/z*. Fragmentation and tandem scanning were performed in a MS3 synchronous precursor ions selection conditioned by mass difference between the fragment ions of either ΔM = 114.0429 *u* (corresponding to the ubiquitin tag GG) or ΔM = 383.2281 *u* (corresponding to the ubiquitin tag LRGG), both detected with an asymmetric mass tolerance of −3 ppm/+7 ppm. Only two (N = 2) ions were allowed for synchronous selection in the MS3 mode, provided that the mass difference between ions in pair was registered.

Liquid chromatography separation was accomplished on an Ultimate 3000 RSLCnano (Thermo Scientific, Rockford, IL, USA). Samples were loaded onto an enrichment Acclaim µ-Precolumn (0.5 mm × 3 mm, 5 µm) (Thermo Scientific, Rockford, IL, USA) at a flow rate of 15 µL/min for 3.5 minutes in 2% acetonitrile, supplied by 0.1% formic acid and 0.03% acetic acid. Analytical separation was carried out at a flow rate 0.3 µL/min using an Acclaim Pepmap^®^ C18 (75 µm × 150 mm, 2 µm) (Thermo Scientific, USA) column in a gradient of mobile phase A (water with 0.1% formic acid and 0.03% acetic acid) and mobile phase B (acetonitrile with 0.1% formic acid and 0.03% acetic acid) in the following gradient: 2%–37% of mobile phase B for 45 min, followed by column washing in 90% of mobile phase B for 8 min, with equilibration of the column under initial gradient conditions (2% of mobile phase B) for 15 min before starting the next run.

Every mass spectrometry experiment was performed using pooled brain mitochondrial fractions isolated from two mice. For each group of animals, four independent experiments were carried out.

### 2.5. Protein Identification and GO Annotation

Raw data files were converted in MGF-files using MSConvert (Proteowizard, Palo Alto, CA, USA). Peak lists obtained from converted spectra were identified using X!Tandem Vengeance, version (2015.12.15.2). The search was conducted using SearchGUI, version 3.3.0 [22,23].

Protein identification was conducted against a concatenated target/decoy version of the *Mus musculus* (16,915 > 99.9%) complement of the UniProtKB fasta file (release February 2018). The decoy sequences were created by reversing the target sequences in SearchGUI. The identification settings were as follows: trypsin (specific), with a maximum of three missed cleavages within ±5.0 ppm tolerance at MS1 level, and ±0.03 Da tolerance for MS2 tolerances. The following variable modifications were set: carbamidomethylation of C (+57.021464 Da), oxidation of M (+15.994915 Da), ubiquitination of K as GG-tag (+114.042927 Da), and long ubiquitination tag of K (+383.228102 Da). Variable modifications refined after the search procedure. Peptides and proteins were inferred from the spectrum identification results using PeptideShaker version 1.16 (Compomics, Gent, Belgium). Peptide spectrum matches (PSMs), peptides, and proteins were validated at a 1.0% false discovery rate (FDR) estimated using the decoy hit distribution.

Proteins were classified by their cellular localization, molecular functions, and biological process involvement in terms of Gene Ontology (GO) annotations using STRAP software (version 1.5.0.0) [24].

Each protein listed in the Tables and the Supplementary Materials was identified at least in three independent experiments, each of which employed independent brain mitochondrial samples, as well as their chromatographic and proteomic processing.

## 3. Results

In accordance with results of our recent research [17,25], a single dose administration of MPTP (30 mg/kg, i.p.) caused a pronounced decrease (−76 ± 6%) in the locomotor activity of mice evaluated in this study in the open field test. Pretreatment with isatin (100 mg/kg, i.p.) significantly (*p* < 0.05) improved the locomotor activity, which however, was lower than in control animals (−44 ± 11%). Isatin administered to intact mice also decreased locomotor activity (−33 ± 8%). This effect may be attributed to known sedative activity as described in the literature (see for review, [26]).

Proteomic profiling of the brain mitochondrial fraction of the control mice resulted in reliable identification of 565 proteins (see Supplementary Materials). Among them, 301 proteins were annotated by GO as mitochondrial proteins. These included both intrinsic mitochondrial proteins, which localized in the inner mitochondrial membrane and matrix, and also proteins of the outer mitochondrial compartment, as well as proteins from extra-mitochondrial compartments associated with mitochondria. Good evidence exists that many extra-mitochondrial proteins interact with mitochondrial membranes, and some of them (e.g., histones [27,28]) are even inserted in the mitochondrial membranes. In this context, it is relevant to consider all of the identified proteins either as intrinsic mitochondrial proteins (i.e., proteins of the inner mitochondrial membrane and matrix) or as mitochondria associated proteins, including mitochondrial proteins of the outer mitochondrial membrane and of the intermembrane space. These mitochondria-associated proteins can be thus defined as proteins of the outer mitochondrial compartment. Such subdivision is important in the context of mitochondrial ubiquitination, because certain evidence exists that outer mitochondrial proteins and proteins of the outer surface of the inner mitochondrial membrane can be ubiquitinated by the cytosolic ubiquitin conjugation machinery [29,30]. Moreover, it appears that even UCP2 (mitochondrial uncoupling protein 2), which is located in the inner mitochondrial membrane [31], can be ubiquitinated by the cytosolic ubiquitin conjugating machinery [32].

**Figure 1:**
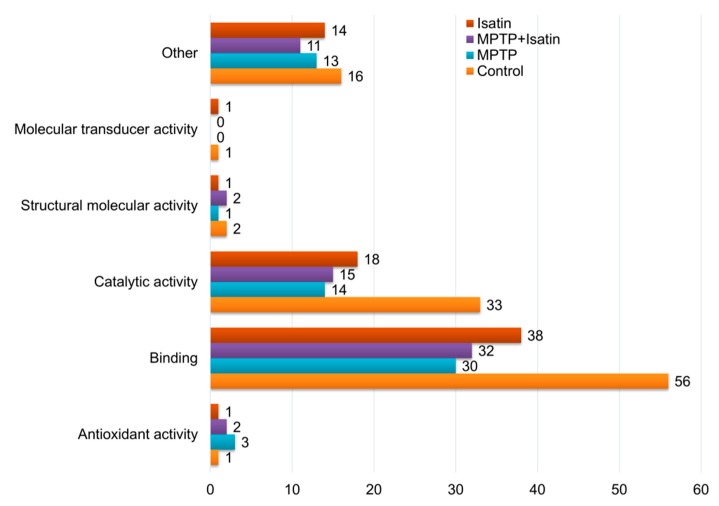
Distribution of identified ubiquitinated proteins among GO groups. Numbers designate the number of identified ubiquitinated proteins in each experimental group of animals.

**Figure 2:**
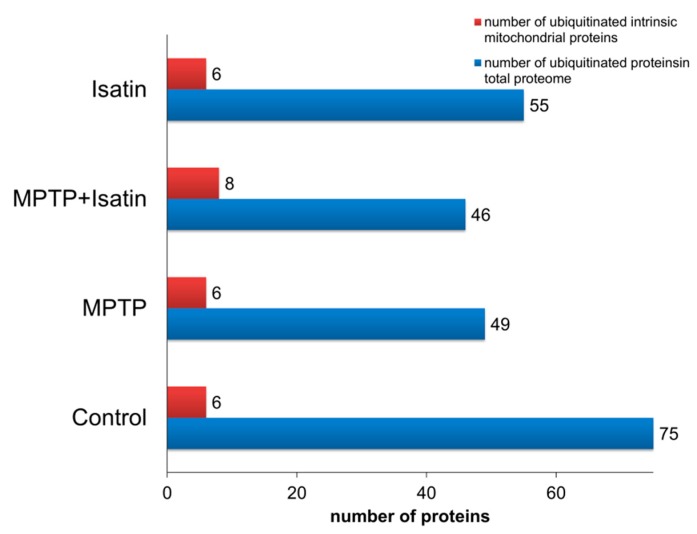
Total number of ubiquitinated proteins identified in the mouse brain mitochondrial fraction.

With this assumption, the brain mitochondrial fraction of control mice contained at least 75 individual ubiquitinated proteins; the number of the ubiquitinated sites in them varied from 1 to 4 (Figure 1; Supplementary Materials). In a pilot experiment, preincubation of brain mitochondrial lysates with the deubiquitinase inhibitor degrasyn (WP-1130; final concentration 10 µM) for 30 min at 37 °C had no influence of the profiles of the ubiquitinated proteins. This suggests that under our experimental conditions, endogenous deubiquitinases had insignificant impact on the content of ubiquitinated proteins in the brain mitochondrial fraction. Among these 75 proteins, only six proteins can be defined as proteins of the inner mitochondrial compartments. Their proportion in the studied groups varied from 8% (in control) to 17% (MPTP+isatin) (Figure 2). According to the GO annotation, they belonged to several functional groups (Table 1). In the context of molecular functions, ubiquitinated mitochondrial proteins of control animals were preferentially involved in catalytic and binding functions. 

MPTP administration to mice decreased the number of ubiquitinated proteins (*n* = 49; Figure 2). The list of ubiquitinated proteins identified in the brain mitochondrial fraction of MPTP-treated mice changed qualitatively, and contained only five proteins that were identified in intact animals. Poor correspondence between identified proteins from control and MPTP-treated mice suggested that the development of MPTP-induced toxicity had a significant impact on the ubiquitination of proteins associated with mitochondria (Figure 1, Table 1). Pretreatment of mice with isatin not only reduced manifestations of MPTP-induced neurotoxicity, but also influenced the profiles of all ubiquitinated proteins detected in the crude mitochondrial fraction of the mouse brain. The total number of ubiquitinated proteins in the crude mitochondrial fraction of the mouse brain and their molecular functions remained basically the same as in the crude mitochondrial fractions of the MPTP-treated mice (Table 1). However, despite similarity in molecular functions, ubiquitinated proteins in the fraction differed qualitatively as compared with both the control and the group of MPTP-treated mice (Table 1, Supplementary Materials).

Isatin administration to intact mice also caused significant changes in both the number of identified proteins in the fraction, as well as the profile of ubiquitinated proteins (Table 1, Supplementary Materials). The total number of mitochondrial proteins was lower than in both the control group and the group of MPTP-treated mice, and the same as in the group of mice treated with both MPTP and isatin. The profile of ubiquitinated proteins identified in the crude mitochondrial fraction of isatin-treated mice (*n* = 55; Figure 2) was also rather specific (Table 1, Supplementary Materials).

In the case of so-called intrinsic mitochondrial proteins, their ubiquitination profiles were highly specific and lacked any common components for all four groups of animals (Table 1).

It should be noted that the crude mitochondrial fraction of the mouse brain was isolated within 2 h after administration of MPTP and isatin to mice. Consequently, the changes in the ubiquitination state of mitochondrial proteins obviously reflected immediate short-term biological responses to these treatments that did not involve long-term adaptive mechanisms (mitochondrial biogenesis etc.).

Besides ubiquitination, we have also evaluated the oxidative status of mitochondrial proteins by the presence of oxidized methionine (see the Materials and Methods section). Although the number of mitochondrial proteins containing oxidized methionine residue(s) in the control group was somewhat higher, analysis of proteins carrying both the ubiquitin signature and oxidized methionine revealed very interesting tendencies. The percent of mitochondrial proteins containing both modifications increased in the following order: control (8% of total ubiquitinated proteins) > MPTP (12%) > MPTP + isatin (15%) > isatin (22%) (Supplementary Materials).

From comparison of ubiquitinated proteins associated with the mouse brain mitochondrial fraction (Supplementary Materials) with mouse brain mitochondrial proteins specifically bound to the proteasome ubiquitin receptor, the Rpn10 subunit [17], did not reveal any common proteins. This raises the possibility that ubiquitination of mitochondrial proteins is not directly linked to degradation in proteasomes.

## 4. Discussion

Ubiquitination plays an important role in targeting oxidized, misfolded, and damaged proteins from different intracellular compartments for subsequent proteasomal degradation [12,13,14,15]. This process is also important for protein modifications that are unrelated to proteasomal degradation but which are related to other processes, including the regulation of various cell functions [33,34]. Various (patho)physiological conditions, especially mitochondrial dysfunction, have a significant impact on UPS functioning [10,11].

In the context of MPTP-induced Parkinsonism, mitochondria are especially important “players” as the outer mitochondrial membrane enzyme, MAO B, catalyzes the conversion of MPTP in the active toxin, MPP^+^, which inhibits complex I of the respiratory chain, thus creating the conditions for the development of mitochondrial dysfunction [2,3,4,5]. In turn, the latter influences UPS [10,11] and promotes an increased accumulation of ubiquitin immunoreactivity in target cells [11] thus suggesting the formation of aggregates containing ubiquitinated proteins.

It should be noted that the analysis of the ubiquitination state of mitochondrial proteins is complicated by the existence of two mitochondrial compartments: (i) the outer compartment, which includes the outer mitochondrial membrane and the intermembrane space; (ii) the inner mitochondrial compartment, including the inner surface of the inner mitochondria membrane and the matrix. The outer mitochondrial compartment also contains numerous extramitochondrial proteins [31,32]. Such proteins can be ubiquitinated by the extramitochondrial ubiquitination machinery that are bound to mitochondrial membranes [35,36]. In the context of possible ubiquitination, these compartments are not identical. Proteins of the outer compartment (and even the inner mitochondrial membrane) can be ubiquitinated by the extramitochondrial ubiquitinating machinery [29,30,32]. Ubiquitination of proteins located in the inner compartment may obviously involve their own mitochondrial ubiquitin conjugation system, which still remains poorly investigated.

The results of our study indicate that ubiquitination of proteins located in the inner mitochondrial compartment covers just a few proteins (Supplementary Materials, Table 1), representing about 10% of the total pool of ubiquitinated proteins associated with brain mitochondria. Nevertheless, identification of such mitochondrial matrix proteins as Succinate-CoA ligase (EC 2.6.1.13), a tricarboxylic cycle enzyme that is responsible for Succinate-CoA conversion to succinate during substrate-coupled phosphorylation (with GTP formation), and other mitochondrial matrix enzymes listed in the table, provide convincing evidence for ubiquitination of intrinsic mitochondrial proteins by the intrinsic mitochondrial ubiquitin-conjugating machinery (Table 2).

Since ubiquitination profiles of intrinsic mitochondrial proteins are highly specific and involve only several individual proteins, it seems unlikely that their ubiquitination may be attributed to mitochondrial damage (and the involvement of the extramitochondrial ubiquitin conjugation machinery). This is consistent with previous results of in vitro studies: using biotinylated ubiquitin, we also detected the ubiquitination label in a few intramitochondrial brain proteins [20]. This proportion of mitochondrial ubiquitinated proteins corresponds to the results of bioinformatic analysis of human ubiquitinated proteins [37]: mitochondrial ubiquitinated proteins represent about 8% of the total pool of ubiquitinated proteins found in humans. The recent study performed using purified yeast mitochondria also revealed 36 ubiquitinated matrix proteins [38]. However, the mechanisms of their ubiquitination still remain poorly characterized. Thus, ubiquitination of intrinsic mitochondrial proteins of the brain can contribute to, but cannot determine the overall effect of the neurotoxin MPTP and the neuroprotector isatin on the overall ubiquitination profile of mitochondrial proteins.

A single dose administration of MPTP to mice significantly changed the profile of brain mitochondrial ubiquitinated protein. Since increased ubiquitin immunoreactivity is registered in the brain of experimental animals only after prolonged treatments with MPTP [11], the altered repertoire of endogenously ubiquitinated proteins observed within 2 h after a single dose of MPTP administration may be thus be considered as the earliest reaction of the ubiquitin conjugation machinery to the toxin. Interestingly, some of the ubiquitinated proteins associated with the brain mitochondrial fraction of MPTP-treated mice are involved in neurodegeneration (see Table 3).

**Figure 3:**
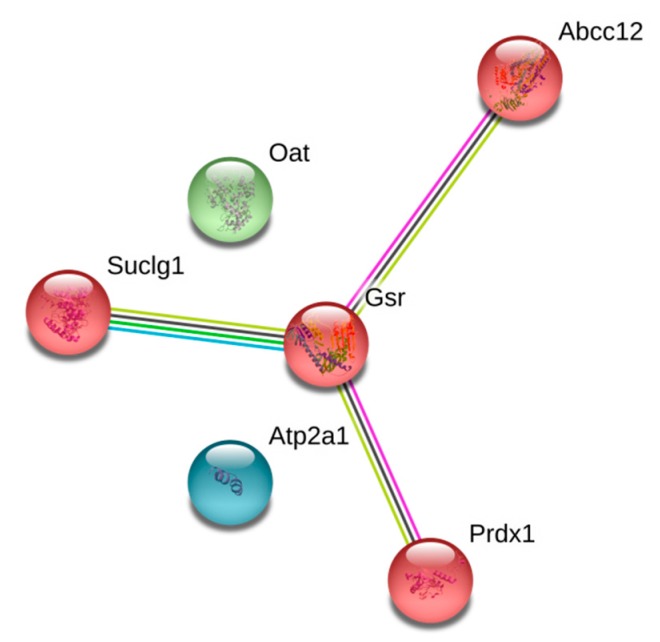
Functional links between mitochondrial ubiquitinated proteins of MPTP-treated mice. The links have been composed by the STRING database resource. Abbreviations used by the STRING resource designate the following proteins: Abcc12—multidrug resistance-associated protein 9; Atp2a1—sarcoplasmic/endoplasmic reticulum calcium ATPase 1; Gsr—mitochondrial glutathione reductase; Oat—mitochondrial ornithine aminotransferase; Prdx1—peroxiredoxin-1; Suclg1—mitochondrial succinate-CoA ligase (ADP/GDP-forming) subunit alpha. The links have been generated using the STRING high confidence score of 0.7. Other explanations are given in the text.

In the context of ubiquitination of intrinsic mitochondrial proteins, it should be noted that they were functionally linked only in the brain mitochondria of MPTP-treated mice (Figure 3).

In these functional links, glutathione reductase (Gsr), catalyzing the reaction of NADPH-dependent glutathione reduction, was the core element (Figure 3). According to STRING database, Gsr has functional links with (Figure 3): (i) succinate CoA ligase, catalyzing substrate level phosphorylation reaction in the Krebs cycle, (ii) peroxiredoxin 1, the protein involved in the antioxidant defense system; (iii) multidrug resistance-associated protein 9, a novel member of the multidrug resistance-associated protein (MRP) family, contributing to decreased drug accumulation [67]. Peroxiredoxin 1 ubiquitination was detected in endothelial cells during ischemic insult; this targeting of peroxiredoxin 1 for degradation deteriorates ischemic brain damage [68]. Such links were not observed in the groups of animals treated with either MPTP or isatin, or only with isatin, due to different patterns of the mitochondrial ubiquitinated proteins. This suggests that the neuroprotector effect of isatin may be associated with blockade of functional links involving ubiquitinated proteins targeted for subsequent degradation.

It is known that the outer mitochondrial membranes and the proteins of the outer mitochondrial compartment are involved in numerous interactions between mitochondria and mitochondria associated membranes (MAM) [69]. Such interactions are crucial for various important cell functions, including mitochondrial morphology, apoptosis, autophagy, Ca^2+^ signaling, endoplasmic reticulum(ER)–mitochondria tethering, ER stress signaling [69]. In this context, the altered repertoire of ubiquitinated proteins determined in the brain mitochondrial fraction of MPTP-treated mice obviously reflects the impaired interactions between the mitochondria and the extra-mitochondrial compartments (MAM).

It is known that MPTP administration induces the development of oxidative stress, accompanied by oxidation of brain proteins [70]. Our study has shown that the repertoire of ubiquitinated proteins from the brain mitochondria of MPTP-treated mice includes oxidized proteins. Interestingly, their proportion was somewhat higher in the control, while the proportion of ubiquitinated and oxidized proteins increased in the following order: control > MPTP > MPTP + isatin > isatin. This suggests that the neuroprotector effect of isatin could be partially attributed to increased ubiquitination of oxidized mitochondrial proteins.

Isatin (indoledione-2,3) is an endogenous neuroprotector which is found in mammalian brain, peripheral tissues, and body fluids [26]. Besides the reversible inhibition of MAO B, which can account for the decreased bioactivation of MPTP, isatin interacts with numerous isatin-binding proteins located in various subcellular organelles, including mitochondria [25,26]. Previous proteomic profiling of brain isatin-binding proteins revealed several enzymes which were directly involved in UPS functioning [17]. However, isatin administration to mice significantly restricted the repertoire of brain mitochondrial proteins bound to the ubiquitin receptor, the 19S Rpn 10 subunit [17]. Moreover, proteomic profiles of ubiquitinated mitochondrial proteins identified in this study (Supplementary Materials) and mitochondrial proteins specifically bound to the proteasome ubiquitin receptor, the Rpn 10 subunit [17], lack any common elements. In addition, some isatin derivatives act as proteasome inhibitors [71,72]. Taken together, all these results suggest that besides increased ubiquitination of oxidized mitochondrial proteins, isatin reduces the possibility of their Rpn10-receptor mediated entry to, and subsequent degradation in proteasomes. Thus, it appears that the neuroprotector effect of isatin can be partially attributed to the switch of proteasomal degradation to selective autophagy, which includes a proteasome-independent route of ubiquitinated proteins [73,74].

## 5. Conclusions

**Figure 4:**
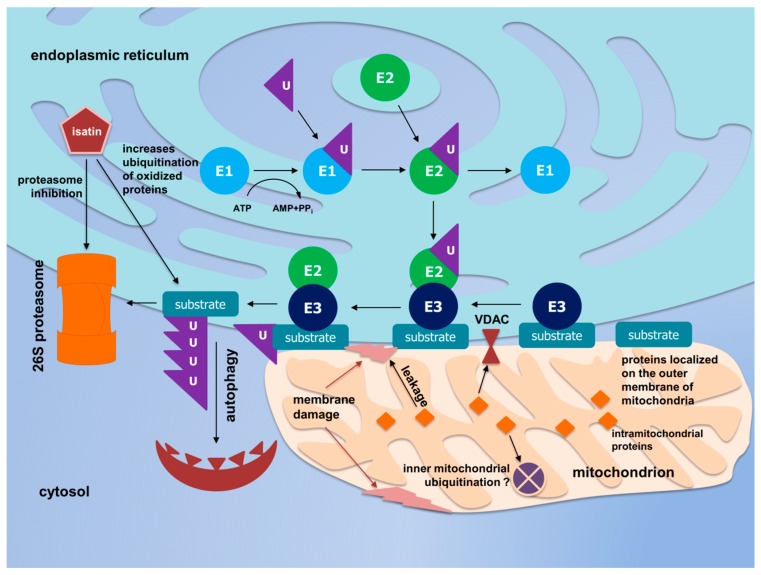
The scheme illustrating the proposed mechanism of neuroprotector action of isatin on metabolic routes of ubiquitinated proteins under conditions of MPTP-induced neurotoxicity. Isatin increases the ubiquitination of oxidized proteins associated with mitochondria, restricts binding of the proteins to the 26S proteasome ubiquitin receptor (Rpn10) and, thus, restricts the access of ubiquitinated proteins to the proteasome and shifts the flux of oxidized and ubiquitinated proteins to autophagy routes.

Administration of MPTP to mice caused locomotor impairments typical of Parkinson’s disease. This was accompanied by dramatic changes in the profile of mitochondrial ubiquitinated proteins. Pretreatment of mice with the neuroprotector isatin not only reduced manifestations of MPTP-induced neurotoxicity, but also influenced the profiles of all ubiquitinated proteins detected in the crude mitochondrial fraction of the mouse brain and increased the proportion of oxidized proteins carrying the ubiquitination signature. Since crude mitochondrial fraction of the mouse brain was isolated within 2 h after administration of MPTP and isatin, the changes in the ubiquitination state of mitochondrial proteins obviously reflect immediate short-term biological responses to these treatments that do not involve long-term adaptive mechanisms (mitochondrial biogenesis etc.). Figure 4 summarizes results of our studies.

Results of our study indicate that ubiquitination of proteins located in the inner mitochondrial compartment covers just a few proteins, and major changes in the ubiquitination state occur in the outer mitochondrial compartment. Earlier we found that isatin also restricted the repertoire of mitochondrial proteins bound to the ubiquitin receptor, the 19S Rpn 10 subunit [17], directly participating in the proteasomal machinery. In addition, mitochondrial ubiquitinated proteins and mitochondrial Rpn10-binding proteins lack any common elements. This raises the possibility that ubiquitination of mitochondrial proteins is not directly linked to degradation in proteasomes. It is known in the literature that some isatin derivatives act as proteasome inhibitors [71,72]. In this context, the increased proportion of oxidized mitochondrial proteins carrying the ubiquitin signature may reflect their impaired proteasomal degradation. In this case the neuroprotector effect of isatin can be (at least partially) attributed to the switch of proteasomal degradation to selective autophagy, which includes a proteasome-independent route of ubiquitinated proteins [73,74].

## Figures and Tables

**Table 1:** Functional annotation of ubiquitinated proteins of mouse brain mitochondria in GO terms.

Accession Number	UniProt ID	Recommended Protein Name	Sample Type				Extramitochondrial Compartment	Intramitochondrial Compartment	Molecular Function			
			Control	MPTP	MPTP+isatin	Isatin			Antioxidant Activity	Binding	Catalytic Activity	Other
CATD_MOUSE	P18242	Cathepsin D			●		●				●	
APR_MOUSE	Q9JM54	Phorbol-12-myristate-13-acetate-induced protein 1			●		●					
TAP1_MOUSE	P21958	Antigen peptide transporter 1	●				●			●	●	●
AKT1_MOUSE	P31750	RAC-alpha serine/threonine-protein kinase	●				●			●	●	●
KPYM_MOUSE	P52480	Pyruvate kinase PKM	●				●			●	●	
ACSS3_MOUSE	Q14DH7	Acyl-CoA synthetase short-chain family member 3, mitochondrial	●					●		●	●	
AL4A1_MOUSE	Q8CHT0	Delta-1-pyrroline-5-carboxylate dehydrogenase, mitochondrial	●					●		●	●	
NALP5_MOUSE	Q9R1M5	NACHT, LRR and PYD domains-containing protein 5	●				●			●		
AT2A1_MOUSE	Q8R429	Sarcoplasmic/endoplasmic reticulum calcium ATPase 1		●		●	●			●	●	
SUCA_MOUSE	Q9WUM5	Succinate-CoA ligase (ADP/GDP-forming) subunit alpha, mitochondrial		●		●		●		●	●	
OAT_MOUSE	P29758	Ornithine aminotransferase, mitochondrial		●				●		●	●	
MRP9_MOUSE	Q80WJ6	Multidrug resistance-associated protein 9		●			●			●	●	
DLDH_MOUSE	O08749	Dihydrolipoyl dehydrogenase, mitochondrial			●			●		●	●	●
CATB_MOUSE	P10605	Cathepsin B			●		●			●	●	
CLH1_MOUSE	Q68FD5	Clathrin heavy chain 1			●		●			●		
ADCYA_MOUSE	Q8C0T9	Adenylate cyclase type 10			●		●			●	●	
FPPS_MOUSE	Q920E5	Farnesyl pyrophosphate synthase			●		●			●	●	
AATM_MOUSE	P05202	Aspartate aminotransferase, mitochondrial				●		●		●	●	
ERAL1_MOUSE	Q9CZU4	GTPase Era, mitochondrial				●		●		●		
TO20L_MOUSE	Q9D4V6	TOMM20-like protein 1				●	●			●		●
PRDX1_MOUSE	P35700	Peroxiredoxin-1		●	●	●	●		●	●		
GSHR_MOUSE	P47791	Glutathione reductase, mitochondrial		●				●	●	●		●

Notes: color shows (extra) mitochondrial localization of the proteins.

**Table 2:** Intrinsic brain mitochondrial proteins and their ubiquitination sites.

UniProt ID	Protein Name	Gene Name	Sequence	Ubiquitination	Oxidized Residue	Confidence, %
O08749	Dihydrolipoyl-dehydrogenase, mitochondrial	Dld	IPVNNRFQTKSTDR	K446		98.99
P29758	Ornithine aminotransferase, mitochondrial	Oat	LFNYNKVLPMNTGVEAGETACK	K135	M139	93.59
Q14DH7	Acyl-CoA synthetase short-chain family member 3, mitochondrial	Acss3	TPPPGQAGK	K472		89.51
Q8CHT0	Delta-1-pyrroline-5-carboxylate dehydrogenase, mitochondrial	Aldh4a1	NESVGYYVEPCIIESKDPQEPIMK	K437		95.86
Q9CZU4 *	GTPase Era, mitochondrial	Eral1	LNPQVLQCLTKFSQVPSILVLN	K225		93.35
Q9WUM5	Succinate-CoA ligase [ADP/GDP-forming] subunit alpha, mitochondrial	Suclg1	KAKPVVSFIAGITAPPGR	K280		100.00
P05202	Aspartate aminotransferase, mitochondrial	Got2	GINVCLCQSYAKNMGLYGER	K279		93.83
P47791	Glutathione reductase, mitochondrial	Gsr	RDAYVSRLNTIYQNNLTK	K141		92.89
P35700	Peroxiredoxin-1	Prdx1	GSDTIKPDVNK	K185		99.48

Notes: *—long chain ubiquitination tag (LRGG).

**Table 3:** Brain proteins ubiquitinated in mice treated with MPTP and their involvement in neurodegeneration.

UNIPROT Accession Number	Protein Name	Involvement in Neurodegeneration	Reference
Q6ZPY5	Zinc finger protein 507	Alterations in ZNFs are involved in the development of neurodegeneration	[39]
Q91YE6	Importin-9	Regeneration of injured neurons	[40]
P68368	Tubulin alpha-4A chain	Alpha-tubulin levels decreased mainly in neurons containing neurofibrillary tau pathology	[41]
Q9QZ04	MAGE-like protein 2	MAGE proteins form complexes with E3 ubiquitin ligases	[42]
Q9R0G7	Zinc finger E-box-binding homeobox 2	It is involved in the regulation of microRNA in glioma stem cells	[43]
Q8BZ36	RAD50-interacting protein 1	It functions as a multitask protein, and is involved in genomic stability, ER homeostasis, and autophagy	[44]
Q8CE72	Protein JBTS17	Jbts17 mutant mice have cilia transition zone defects and related cerebellar anomalies	[45]
Q6P7F1	MAGUK p55 subfamily member 4	Plays a role in several CNS disorders	[46]
P43300	Early growth response protein 3	Gene encoding this protein is induced by alpha-synuclein	[47]
Q8C4A5	Putative Polycomb group protein ASXL3	Is upregulated in Alzheimer’s disease	[48]
Q9ESK9	RB1-inducible coiled-coil protein 1	Its insufficiency causes neuronal atrophy and is involved in the pathology of Alzheimer’s diseases	[49]
P47791	Glutathione reductase, mitochondrial	Is implicated in glutathione reduction. GSH is important for pathogenesis of Parkinson’s disease	[50,51]
Q9CR16	Peptidyl-prolyl *cis-trans* isomerase D	Binds to hyperphosphorylated Tau proteins in degenerating neurons	[52]
Q9EQK5	Major vault protein	Is implicated in senescence-associated apoptosis resistance	[53]
Q3UUG6	TBC1 domain family member 24	Truncating mutation results in severe neurodegeneration	[54]
P23950	mRNA decay activator protein ZFP36L1	Involved in mRNA stability in the human brain	[55]
P97500	Myelin transcription factor 1-like protein	Influences memory-related processes	[56]
Q9QZQ1	Afadin	Maintenance of dendritic structure and excitatory tone	[57]
P81117	Nucleobindin-2	Altered levels found in neuropsychiatric disorders	[58]
P58006	Sestrin-1	A negative feedback regulator of TOR; its loss results in various TOR-dependent, age-related pathologies	[59]
Q9D2H8	Fibronectin type III domain-containing protein 8	Its expression is decreased in patients with PD with dementia	[60]
Q8C079	Striatin-interacting protein 1	Involved in the targeting, attachment, and cytoskeletal transport of autophagosomes, which are accumulated in neurodegenerative neurons	[61]
Q8BJQ2	Ubiquitin carboxyl-terminal hydrolase 1	Undergoes oxidative modification in both Alzheimer’s disease and Parkinson’s disease	[62]
Q91WJ8	Far upstream element-binding protein 1	Being a substrate for ubiquitination by Parkin, it plays an important role in development of Parkinson disease	[63]
P29758	OAT, mitochondrial	Ornithine aminotransferase deficiency causes gyrate atrophy	[64]
P35700	Peroxiredoxin-1	Plays a protective role in counteracting Aβ injury by increasing cell viability preserving neurites, and decreasing cell death	[65]
Q8C4S8	DENN domain-containing protein 2A	DENN proteins regulate autophagy	[66]

## Supplementary Materials

The Supplementary materials are available as a Microsoft Excel file, available online at http://www.mdpi.com/2073-4409/7/8/91/s1. It contains information about the whole proteomic profiles of crude mitochondrial fractions from all groups of animals, lists of ubiquitinated proteins, oxidized proteins, and ubiquitinated and oxidized proteins.
